# Periconceptional Folic Acid Supplementation Benefit to Development of Early Sensory-Motor Function through Increase DNA Methylation in Rat Offspring

**DOI:** 10.3390/nu10030292

**Published:** 2018-03-01

**Authors:** Wen Li, Zhenshu Li, Shou Li, Xinyan Wang, John X. Wilson, Guowei Huang

**Affiliations:** 1 Department of Nutrition and Food Science, School of Public Health, Tianjin Medical University, 22 Qixiangtai Road, Heping District, Tianjin 300070, China; liwen828@tmu.edu.cn (W.L.); lizhenshu@tmu.edu.cn (Z.L.); lishou@tmu.edu.cn (S.L.); wangxinyan@tmu.edu.cn (X.W.); 2 Department of Exercise and Nutrition Sciences, School of Public Health and Health Professions, University at Buffalo, Buffalo, NY 14214-8028, USA; jxwilson@buffalo.edu

**Keywords:** maternal folic acid, neurobehavioral development, DNA methylation, offspring

## Abstract

Periconceptional maternal folate levels may alter DNA methylation patterns and health outcomes in offspring. We hypothesized that maternal folic acid supplementation alters fetal neural development through DNA methylation in the fetal brain. Twenty-eight rats were randomly assigned to four groups: three groups of the female rats were fed folate-normal, folate-deficient or folate-supplemented diets from seven days before mating to delivery. In another group, folic acid supplementation diet short-period group was fed a folate-normal diet, except for 10 days (begin mating) when this group was fed a folate-supplemented diet. After delivery, the diets were changed to folate-normal diet for all four groups. The cliff avoidance and forelimb grip tests were used to assess sensory motor function of rat offspring. The results indicate that maternal folic acid supplementation improved the early development of sensory-motor function in offspring. Maternal folic acid supplementation increased the methylation potential, global DNA methylation (5-mC) and DNA methyltransferase expression and activity in the brains of the offspring. In conclusion, maternal folic acid supplementation increases DNA methylation pattern in offspring brain and improves the early development of sensory-motor function.

## 1. Introduction

Folate requirements are increased during pregnancy because of the increased abundance of one-carbon transfer reactions for DNA synthesis and methylation reactions [1,2]. Folic acid supplementation at 400 μg per day or higher is recommended worldwide before and during early stages of pregnancy to reduce the incidence of neural tube defects (NTDs) [3]. Additionally, the food supply in many countries is fortified with folic acid to increase folate levels in women of childbearing age and thereby decrease NTDs risk [4]. High maternal folate levels also may have other beneficial effects in offspring, such as reduced risks of low birth weight, language delay, autism, and childhood brain tumors [5,6,7,8,9]. Some health outcomes in offspring may depend on alteration of fetal DNA methylation patterns [10,11,12]. A new DNA methylation pattern is established during embryogenesis soon after implantation and consequently the genome and epigenome of the developing fetus may be highly susceptible to environmental modifiers such as maternal folate levels [13]. 

Our previous research in neural stem cell (NSC) cultures found that folic acid stimulates NSC proliferation by modifying DNA methylation levels [14,15]. Therefore, we hypothesized that maternal folic acid supplementation alters fetal neural development by modulating DNA methylation. 

## 2. Materials and Methods

### 2.1. Rats and Diets

The Tianjin Medical University Animal Ethics Committee approved the experimental protocols of this study (TMUaEC2015001). Mature, three-month-old male and female Sprague-Dawley rats were purchased from Charles River Laboratories, Beijing, China. The rats were housed under 24 ± 2 °C controlled SPF condition with 12-h light/dark cycle and allowed ad libitum access to food and water during the experiment. Five females were housed with each male during mating and then pregnant dams were housed singly. 

The rats were randomly assigned to four groups (seven female rats for each group): (1) folate-deficient diet group (DD group) that was fed a folate-deficient diet from seven days before mating to delivery; (2) folic acid normal diet group (ND group) that was fed a control diet from seven days before mating to delivery; (3) folic acid supplementation diet short-period group (FD-S group) that was fed a control diet from seven days before mating to delivery except for a 10-day-long period (beginning with mating) when this group was fed a folic acid supplementation diet; (4) folic acid supplementation diet long-period group (FD-L group) that was fed a folic acid supplementation diet from seven days before mating to delivery. After delivery, the diets were changed to the control diet for all four groups.

All the diets were purchased from TestDiet (St. Louis, MO, USA). The control diet was AIN-93, which contains 2.1 mg folic acid/kg and has been extensively used in previous animal studies of folate [16,17]. The folate-deficient and folic acid supplementation diets only differed from AIN-93 by containing, respectively, 0.1 and 3.5 mg folic acid/kg. 

Some newborn offspring (pups) were selected randomly from each dam and euthanized, then the blood and the brain samples were collected, and the brain tissue was snap-frozen and stored at −80 °C. The remaining pups were used for neurobehavioral tests and euthanized at four months of age.

### 2.2. Cliff Avoidance Test

The cliff avoidance test was evaluated from postnatal day (PD) 4 to PD8 to assess the integration of exteroceptive input (vibrissae) and loco-motor output [18]. Pups were placed on the edge of a platform (20 × 20 × 20 cm) with forepaws and heads extending over the edge. If the pup avoided the cliff and turned backwards then the test result was recorded as positive. If the pup did not make a response within 60 s or fell off the platform then the test result was recorded as negative.

### 2.3. Forelimb Grip Test

The forelimb grip strength test was carried out from PD8 to PD14 to assess neuromuscular function [19]. The duration of forelimb grip of a metal bar (diameter × length = 2 mm × 7.5 cm) was measured using a low-force testing system (Model-RX-5, Aikoh Engineering, Nagoya, Japan). Every pup was tested thrice and its mean grip duration was recorded.

### 2.4. Folate and Homocysteine (Hcy) Concentration

Angular venous blood was collected from pups in coagulant tubes and then centrifuged at 3000× *g* for 10 min to obtain serum. The brain tissues of pups were homogenized by a motor-driven tissue homogenizer (PT1200E; Kinematica, Lucerne, Switzerland). Folate concentration was determined using a competitive protein-binding assay and an automated chemiluminescence system (Immulite 2000 Xpi; Siemens, Berlin, Germany) according to the manufacturer’s instructions. The assay detected all types of folates, including folic acid, dihydrofolate, and tetrahydrofolate [20]. Serum samples were diluted 10 times with 0.9% saline to reach the detection limit of this system, which was 1–24 ng/mL. The total Hcy concentration was quantified using an Auto-Chemistry Analyzer (DIRUI; Changchun, China) and Hcy Reagent (Medical System, Ningbo, China), for which the detection limit was 3–150 μmol/L.

### 2.5. Methylation Potential Assay

*S*-adenosylmethionine (SAM) and *S*-adenosylhomocysteine (SAH) were determined in brain tissue of pups. The brain samples were homogenized by a motor-driven tissue homogenizer (PT1200E, Kinematica, Lucerne, Switzerland) and kept on ice. 100 mg extracts were resuspended in 300 μL of 0.4 mol/L ice-cold perchloric acid. The homogenate was centrifuged at 20,000× *g* for 10 min at 4 °C and the supernatant was collected. The supernatant was filtered through 0.45 μm (Millipore, Billerica, MA, USA) and then was loaded into a Venusil MP-C18 column (250 mm × 4.6 mm, 5 μm particle; Agela Technologies, Wilmington, DE, USA) fitted with a matched guard column, run by a Waters HPLC system (Milford, MA, USA) and connected to an ultraviolet detector. Absorption of eluted compounds was monitored at λ = 254 nm. A two-buffer elution system was used: mobile phase A and B, both contain 10 mmol/L ammonium formate and 4 mmol/L l-heptanesulfonic acid (pH = 4). Mobile phase B contains 50% acetonitrile by volume. Elution of SAM and SAH was achieved at a flow rate of 1 mL/min with the following parameters: 0–0.5 min, 100% A; 0.5–20 min, linear gradient to 75% A and 25% B; 20–30 min, 25% B; 30–45 min, 100% A. Chromatograms were recorded by a Hewlett-Packard HP3394 integrator with its quantification accomplished by automatic peak area integration. SAM and SAH standards were used to identify the elution peaks [16].

### 2.6. DNA Methyltransferase Activity Assay

Nuclear extracts of brain tissue of pups were isolated using the nuclear extraction kit (Merck KGaA, Darmstadt, Germany). DNA methyltransferase (DNMT) activity was measured using a DNMT activity/inhibition assay kit according to the manufacturer’s instructions (Active Motif, Carlsbad, CA, USA). A lot-specific standard curve was created with the DNMT1 provided in the kit. Optical density (OD) was measured on a microplate reader at 450 nm and DNMT activity ((OD/(h·mg)) was calculated according to the following formula

$$\mathrm{DNMT}\;\mathrm{activity}(\mathrm{OD}/h/\mathrm{mg})=\frac{\left(\mathrm{Average}\;\mathrm{Sample}\;\mathrm{OD}-\mathrm{Average}\;\mathrm{Blank}\;\mathrm{OD}\right)}{\mathrm{Protein}\;\mathrm{amount}\;(\mu g)*\times \mathrm{hour}**}\times 1000$$

where,

* Protein amount added into the reaction.

** Incubation time used for the reaction.

### 2.7. Global DNA Methylation Analysis

Nucleic acids were isolated from brain tissues of pups using the Genomic DNA Purification Kit (Promega, Madison, WI, USA) according to the manufacturer’s instructions. Nucleic acid concentrations were determined with a Nucleic acid spectrometer (NanoDrop 2000, Thermo Scientific, Waltham, MA, USA). The level of global DNA methylation was evaluated using a MethylFlash™ Global DNA Methylation (5-mC) ELISA Easy Kit (EpiGentek, Farmingdale, NY, USA). A lot-specific standard curve was created with the positive control (containing 5% 5-mC) provided in the kit. Optical density (OD) was measured on a microplate reader at 450 nm and the level of 5-mC was calculated according to the following formula

$$5-\mathrm{mC}\%=\frac{OD_{\mathrm{sample}}-OD_{\mathrm{negative}}}{\mathrm{slope}\times S*}\times 100\%$$

where, * S is the amount of input sample DNA in ng.

### 2.8. Statistical Analysis

The data were expressed as proportion or mean ± SD according to the data distribution. Comparisons between different groups were performed by one-way ANOVA for normally distributed data, and by Pearson’s chi-squared or Fisher’s exact test for frequency analysis. Multiple timepoint data were compared using the repeated-measures ANOVA. The statistical software package IBM SPSS 16.0 (IBM, Armonk, NY, USA) and SAS 9.4 (SAS Institute, Berkley, CA, USA) was used to evaluate differences within groups, which were considered statistically significant at *p* < 0.05.

## 3. Results

### 3.1. Maternal Folic Acid Had No Effect on Maternal Body Weight or Litter Size

None of the dams showed weight loss, failure to gain weight, or premature death. The overall pattern of weight gain during pregnancy was similar among the four experimental groups and the mean weight gain was 116.42 ± 24.02 g. No significant differences in the average number of pups per litter were observed among the four groups and the average number of pups per litter was 14.36 ± 3.48.

### 3.2. Maternal Folic Acid Supplementation Improved Sensory-Motor Development in Offspring

The cliff avoidance and forelimb grip tests were used to assess sensory motor function of rat offspring. From each diet group, 15 female and 15 male pups were selected randomly (4–5 offspring from each dam) for the cliff avoidance test at PD4-8 and the forelimb grip test at PD8-14. The rates of improvement in the cliff avoidance and forelimb grip tests from PND4 to PND14 tended to differ between diet groups. 

**Figure 1:**
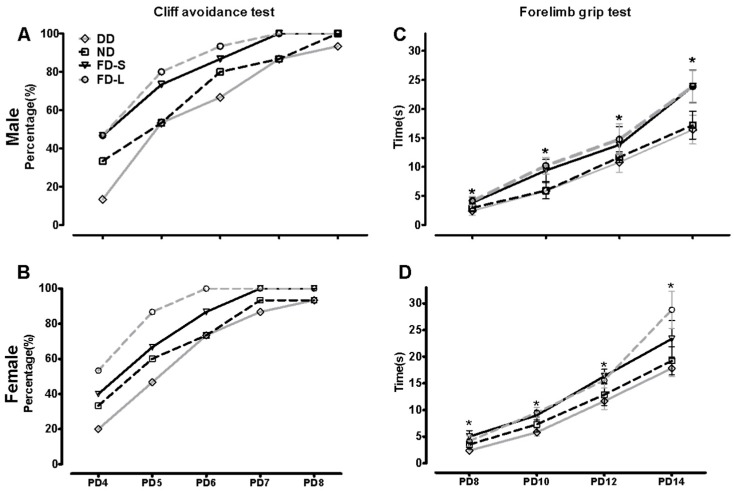
Maternal folic acid supplementation improved sensory-motor development in offspring. Dams were randomly assigned to a folate-normal diet group (ND), a folate-deficient diet group (DD), a folic acid-supplemented diet short-period group (FD-S), and a folic acid-supplemented diet long-period group (FD-L). Thirty pups (15 female and 15 male pups) were selected randomly from each diet group (4–5 offspring from each dam) for the cliff avoidance test at PD4-8 and the forelimb grip test at PD8-14. (**A**) Cliff avoidance test for male offspring (*n* = 15/group); (**B**) Cliff avoidance test for female offspring (*n* = 15/group); (**C**) Forelimb grip test for male offspring (*n* = 15/group); (**D**) Forelimb grip test for female offspring (*n* = 15/group). Data of cliff avoidance test are expressed as proportion, and data of forelimb grip test are expressed as mean ± SD. * Comparing with four groups at α = 0.05/(comparisons times).

For the cliff avoidance test (Figure 1A,B), the folate-deficient (DD) group had the lowest percentages of positive test results both in male and female, although repeated measures analysis showed no statistically significant differences between this group’s results and those of the control (ND) group (male, *t* = 0.15, *p* = 0.878; female, *t* = 0.16, *p* = 0.874), FD-S group (*t* = 0.16, *p* = 0.872) or FD-L group (*t* = 0.11, *p* = 0.914). The highest percentages of positive test results were in the group that had been supplemented throughout pregnancy (FD-L), although there was no significant difference between this group and the group that had been supplemented for the periconception period only (FD-S; *t* = 0.32, *p* = 0.747). 

For the forelimb grip test (Figure 1C,D), the repeated measures analysis showed that the DD group had the shortest grip durations both in male and female (male, *F* = 92.676, *p* < 0.001; female, *F* = 40.425, *p* < 0.001). Folic acid supplementation lengthened grip duration significantly (*F* = 110.249, *p* < 0.001) and supplementation throughout pregnancy was more effective than supplementation limited to the periconception period. Taken together, these results indicate that maternal folic acid supplementation improved the early development of sensory-motor function in offspring.

### 3.3. Maternal Folic Acid Supplementation Increased Folate Level in Pups

**Figure 2:**
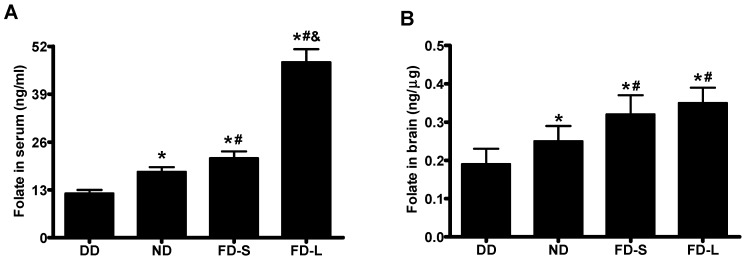
Maternal folic acid supplementation increased folate levels in offspring. Dams were fed as described in Figure 1. (**A**) Serum folate concentration in pups; (**B**) Brain folate concentration in pups. * *p* < 0.05 compared with DD group. # *p* < 0.05 compared with ND group. & *p* < 0.05 compared with FD-S group. Data are expressed as mean ± SD for *n* = 10 pups/group (1–2 pups from each dam).

Serum folate concentrations in offspring (pups) differed significantly between diet groups (*F* = 529.434, *p* < 0.001) and increased in the sequence DD < ND < FD-S < FD-L (Figure 2A). Brain folate concentrations in pups also differed significantly between diet groups (*F* = 15.115, *p* < 0.001) and increased in the same sequence DD < ND < FD-S < FD-L (Figure 2B).

### 3.4. Maternal Folic Acid Supplementation Altered One Carbon Metabolism in Pups

**Figure 3:**
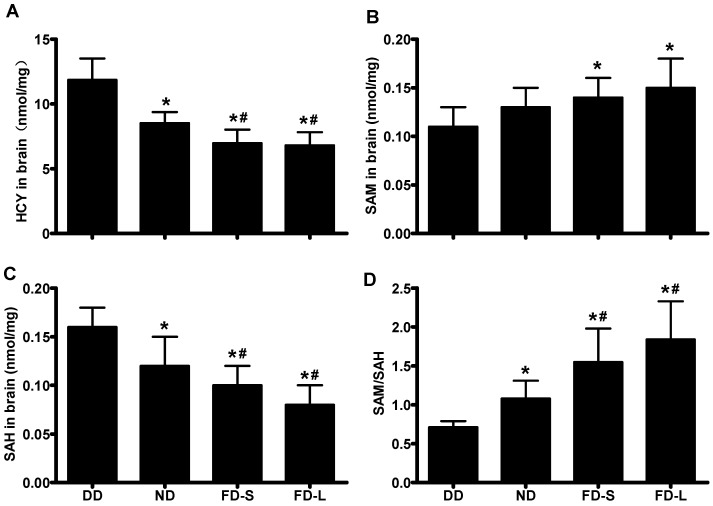
Maternal folic acid supplementation altered one carbon metabolism in offspring. Dams were fed as described in Figure 1. (**A**) Brain Hcy concentration in pups; (**B**) Brain SAM concentration in pups; (**C**) Brain SAH concentration in pups; (**D**) Methylation potential (SAM:SAH ratio) in brain tissue of pups. * *p* < 0.05 compared with DD group. # *p* < 0.05 compared with ND group. Data are expressed as mean ± SD for *n* = 10 pups/group (same pups as Figure 2).

In pups, there were significant differences between the diet groups for brain concentrations of Hcy (*F* = 31.663, *p* < 0.001; Figure 3A), SAM (*F* = 3.749, *p* = 0.022; Figure 3B), SAH (*F* = 21.851, *p* < 0.001; Figure 3C), and brain methylation potential (*F* = 16.223, *p* < 0.001; Figure 3D). Hcy and SAH concentrations decreased in the sequence DD > ND > FD-S > FD-L, whereas SAM concentration and methylation potential increased in the sequence DD < ND < FD-S < FD-L (Figure 3). Taken together, these results indicate that maternal consumption of folic acid affects one carbon metabolism in the brains of their offspring.

### 3.5. Maternal Folic Acid Supplementation Increased Global DNA Methylation Level and DNMT Activity in Offspring Brain

**Figure 4:**
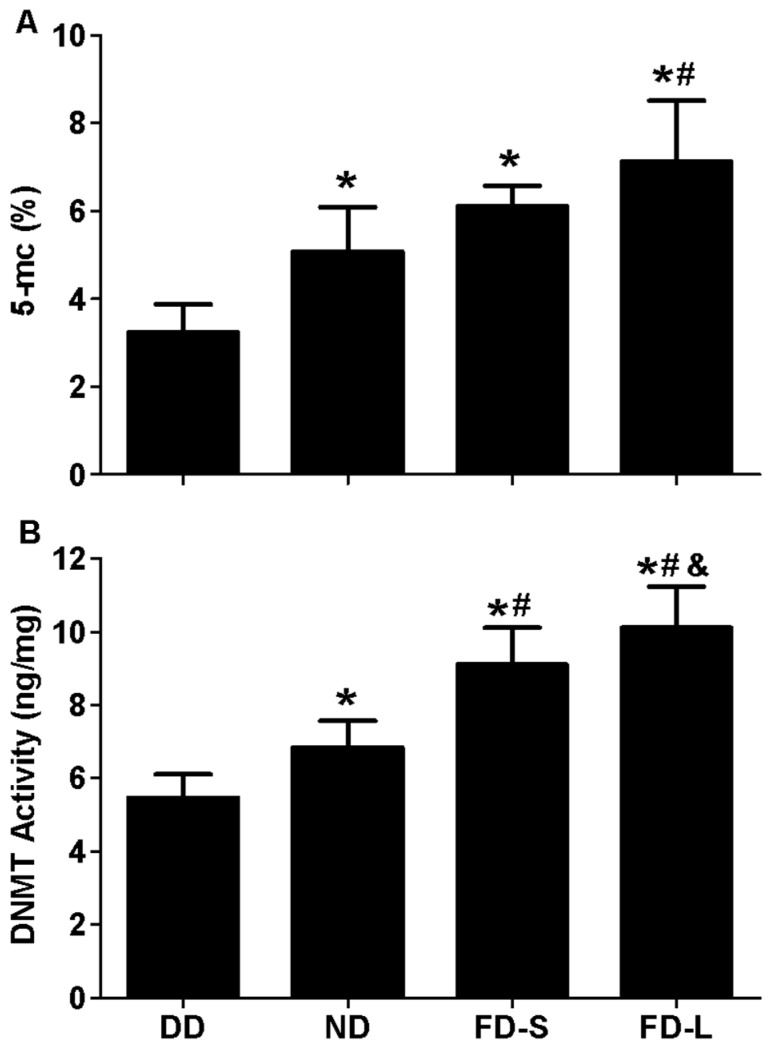
Maternal folic acid supplementation increased global DNA methylation level and DNMT activity in offspring brain. Dams were fed as described in Figure 1. (**A**) Global DNA methylation level (5-mC level) in brain tissue of offspring; (**B**) DNMT activity in brain tissue of offspring. * *p* < 0.05 compared with DD group. # *p* < 0.05 compared with ND group. & *p* < 0.05 compared with FD-S group. Data are expressed as mean ± SD for *n* = 10 pups/group (same pups as Figure 2).

**Figure 5:**
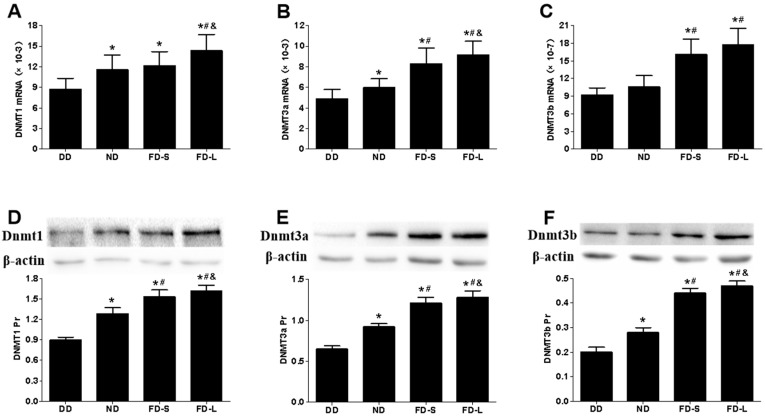
Maternal folic acid supplementation increased the expression of DNMTs in offspring brains. Dams were fed as described in Figure 1. (**A**–**C**) mRNA expression of DNMT1, 3a and 3a in brain tissue of pups; (**D**–**F**) Protein expression of DNMT1, 3a and 3b in brain tissue of pups. * *p* < 0.05 compared with DD group. # *p* < 0.05 compared with ND group. & *p* < 0.05 compared with FD-S group. Data are expressed as mean ± SD for *n* = 10 pups/group (same pups as Figure 2).

In the brains of pups, there were significant differences between the diet groups for global DNA methylation level (5-mC level) (*F* = 8.983, *p* = 0.001; Figure 4A) and DNMT activity (*F* = 44.327, *p* < 0.001; Figure 4B). The 5-mC level was lowest for the DD group, intermediate for the ND and FD-S groups, and highest for the FD-L group. DNMT activity increased in the sequence DD < ND < FD-S < FD-L. Real-time PCR and western blot analyses showed that DNMT1, DNMT3a, and DNMT3b gene and protein expression levels all were lowest in DD group and highest in FD-L group (Figure 5). Taken together, these results indicate that maternal folic acid supplementation increased global DNA methylation level, DNMT activity, and DNMTs expression in the brains of offspring.

## 4. Discussion

The present study found that maternal folic acid supplementation increased in offspring’s brains the methylation potential, global DNA methylation level (5-mC level), DNMT expression and activity, and also improved the early development of sensory-motor function. Evidence is presented that the underlying mechanism may be folic acid-induced modulation of methylation patterns in the offspring brain.

These behavioral tests in this study revealed that maternal folic acid supplementation improved the early development of sensory-motor function in rat offspring. It has been well established in human studies that folic acid supplementation prior to and during early pregnancy can decrease the risk of NTDs [21]. There have also been reports of associations between periconceptional folate consumption and other aspects of human neurodevelopment, such as language delay and autism [6,8,9]. Additionally, positive associations have been found between maternal folate concentrations in early pregnancy and fetal head growth [22], and between maternal folate and children’s mental and social development quotients with children 2 years of age [23]. In our prevent study, the impact of periconceptual folate in adolescent and adult rat offspring also be discussed. The Morris water maze test evaluate learning and memory ability in adolescent (PND45) and adult (PND90) offspring. The results showed that maternal folate deficiency delayed early sensory-motor reflex development, impaired spatial learning, and memory ability of offspring [24].

In this study, three kinds of diets were used, among those diets only folic acid content different, respectively, 0.1, 2.1, and 3.5 mg folic acid/kg. As discussed in our previous study, 3.5 mg folic acid per kilogram diet in rats is equivalent to consumption of a 400 μg folic acid tablet daily on in people consuming a healthy diet [24]. Folic acid as a kind of micromolecule can be getting through placental barrier, so serum folate concentrations in pups increased in the sequence DD < ND < FD-S < FD-L. Folate can also get through blood–brain barrier, so brain folate concentrations in pups also increased in the sequence DD < ND < FD-S < FD-L. The brains of the rat pups in the present study responded to maternal folic acid supplementation with increased in offspring brain the methylation potential, global DNA methylation level (5-mC level), DNMT expression, and activity. According to the developmental origins of health and disease hypothesis, maternal nutrition during gestation can influence the epigenome of the developing fetus and thus modulate physiological outcome. Folate plays a key role in epigenomics and maternal folic acid supplementation produces long-term epigenomic effects in offspring, some of which may be beneficial and others harmful [25,26]. 

The female rats were randomly assigned to four groups, seven female rats for each group. The average number of pups per litter was 14.36 ± 3.48. Because the serum sample is very litter for neonatal rat, the pups which used to test folate euthanized at PD0, and then the blood and the brain samples were collected. The sample size for the test of serum and brain folate was 10 pups/group that mean 1 or 2 pups from each dam. Every dam chose at least one pup. The remaining pups were used for neurobehavioral tests and euthanized at four months of age. Thirty pups (15 female and 15 male pups) were selected randomly from each diet group (4–5 offspring from each dam) for sensory motor function tests. The dada was calculated by sex. The pups were selected randomly and no data was discarded. Although every dam has same parental generation and similar environment; the difference between each littermate was small. Further study should expand the sample size to further verify how maternal folic acid supplementation alters fetal neural development by modulating DNA methylation.

## 5. Conclusions

In conclusion, the present study found that maternal folic acid supplementation modifies DNA methylation pattern in offspring brain. These changes were associated with improvement in the early development of sensory-motor function. These findings are consistent with an epigenomic mechanism by which periconceptional folic acid supplementation benefits neurodevelopment in offspring.
